# Field evaluation of two commercial mosquito traps baited with different attractants and colored lights for malaria vector surveillance in Thailand

**DOI:** 10.1186/s13071-017-2315-1

**Published:** 2017-08-07

**Authors:** Alongkot Ponlawat, Patcharee Khongtak, Boonsong Jaichapor, Arissara Pongsiri, Brian P. Evans

**Affiliations:** 1 Vector Biology and Control Section, Department of Entomology, Armed Forces Research Institute of Medical Sciences (AFRIMS), Bangkok, Thailand; 2 Armed Forces Pest Management Board, Silver Spring, MD USA

**Keywords:** *Anopheles*, BG sentinel, CDC light trap, Colored lights, Attractant

## Abstract

**Background:**

Sampling for adult mosquito populations is a means of evaluating the efficacy of vector control operations. The goal of this study was to evaluate and identify the most efficacious mosquito traps and combinations of attractants for malaria vector surveillance along the Thai-Myanmar border.

**Methods:**

In the first part of the study, the BG-Sentinel™ Trap (BGS Trap) and Centers for Disease Control and Prevention miniature light trap (CDC LT) baited with different attractants (BG-lure® and CO_2_) were evaluated using a Latin square experimental design. The six configurations were BGS Trap with BG-lure, BGS Trap with BG-lure plus CO_2_, BGS Trap with CO_2_, CDC LT with BG-lure, CDC LT with BG lure plus CO_2_, and CDC LT with CO_2_. The second half of the study evaluated the impact of light color on malaria vector collections. Colors included the incandescent bulb, ultraviolet (UV) light-emitting diode (LED), green light stick, red light stick, green LED, and red LED.

**Results:**

A total of 8638 mosquitoes consisting of 42 species were captured over 708 trap-nights. The trap types, attractants, and colored lights affected numbers of female anopheline and *Anopheles minimus* collected (GLM, *P* < 0.01). Results revealed that BGS Trap captured many anophelines but was significantly less than the CDC LT. The CDC LT, when baited with BG-lure plus CO_2_ captured the greatest number of anopheline females with a catch rate significantly higher than the CDC LT baited with BG-lure or CO_2_ alone (*P* < 0.05). The number of anopheline females collected from the CDC LT baited with CO_2_ was greater than the CDC LT baited with BG-lure (646 *vs* 409 females). None of the alternative lights evaluated exceeded the performance of the incandescent light bulb in terms of the numbers of anopheline and *An. minimus* collected.

**Conclusion:**

We conclude that the CDC LT augmented with an incandescent light shows high potential for malaria vector surveillance when baited with CO_2_ and the BG-lure in combination and can be effectively used as the new gold standard technique for collecting malaria vectors in Thailand.

## Background

While the international community has expedited efforts in recent years to control the spread of malaria, the disease remains endemic in 106 countries with one-fifth of the world’s population (1.2 billion people) living in areas with a known high risk of disease transmission [1]. However, within the Greater Mekong Subregion (GMS) of Southeast Asia, an actual decline in malaria incidence and deaths over the previous decade provides some degree of optimism. Regardless, serious concern remains towards the prevalence of multidrug resistance in this region. An added challenge is the presence of “border malaria” cases which has proven exceptionally difficult to monitor and yet poses as a threat to the interior of countries such as Thailand where malaria had been successfully marginalized to the borders several decades prior [2, 3]. Adding to the distress are the complex socio-political issues that have continued to plague the region over decades which only serve to stifle efforts to control the spread of the disease within the region [4]. The situation is further compounded by the fact that all six *Plasmodium* species associated with humans are found within the GMS and additionally, the parasite is vectored by three *Anopheles* species complexes (*An. minimus*, *An. dirus* and *An. maculatus*) with significant ecological variation to be found among and within the complexes [4]. To this end, an effective malaria surveillance program that includes monitoring for human cases, the parasites of interest, and the existence of potential or known vectors is vital to achieving effective health interventions and in evaluating possible impacts on malaria transmission.

Historically, human landing catches (HLCs) were used to survey for the existence of potential vectors of disease and to evaluate the efficacy of vector control operations. Data collected using this technique tended to provide a true sense of potential mosquito vector species and densities since the actual host was the bait. Data from HLCs could also be used to determine entomological inoculation rates, which are true estimates of the disease risk posed to humans [5–7]. However, the ethical issue of placing humans at greater risk for contracting disease, the labor-intensive aspects of the approach and the variation often found among “human attractants” justifies the need for alternative tools that are relatively safer and require minimal man-hours to operate.

To avoid human contact with mosquitoes, various devices have been developed over the years to survey for *Anopheles* mosquitoes [5, 8–12]. The BG Sentinel™ Trap (BGS Trap) (Biogents, A.G. Regensburg, Germany) complemented with the BG lure® (Biogents A.G.) [13], has shown significant promise as a tool for collecting *Ae. aegypti* (L.) [13–16], *Ae. albopictus* (Skuse) [17], *Culex pipiens* L. [15] and *Anopheles* mosquitoes [18–21]. The BG-lure is a dispenser consisting of a blend of mosquito attractants (lactic acid, ammonia, and caproic acid). To date, the BGS Trap has not been field-tested in southeast Asia for anophelines and therefore no information on its efficacy in collecting malaria vectors from Thailand is available. In the current study, we evaluate the efficacy of the BGS Trap (baited with either CO_2_ or the BG-lure or both candidate attractants) compared with the Centers for Disease Control and Prevention (CDC) Miniature light trap Model 512 (CDC LT) (John W. Hock Company, Gainesville, FL, USA) under field conditions in Thailand.

We further evaluate the relative impact of colored lights on the collection of *Anopheles* spp. when used in conjunction with either the CDC or the BGS Trap. Ultraviolet (UV), blue, and green color are visible by insects [22–24]. Red and infrared light are invisible to most insects [24]. However, the majority of mosquito light traps are augmented with incandescent light bulbs which weakly emit the insect visible light spectra [25]. We hypothesized that insect-visible lights (UV, green) would improve the efficacy of mosquito traps by increasing the catch rate relative to invisible or weakly visible lights (incandescent and red). Data from this study may provide preliminary evidence that the incorporation of colored lights into the trap design will lead to an improved surveillance tool.

## Methods

### Study site

**Fig. 1:**
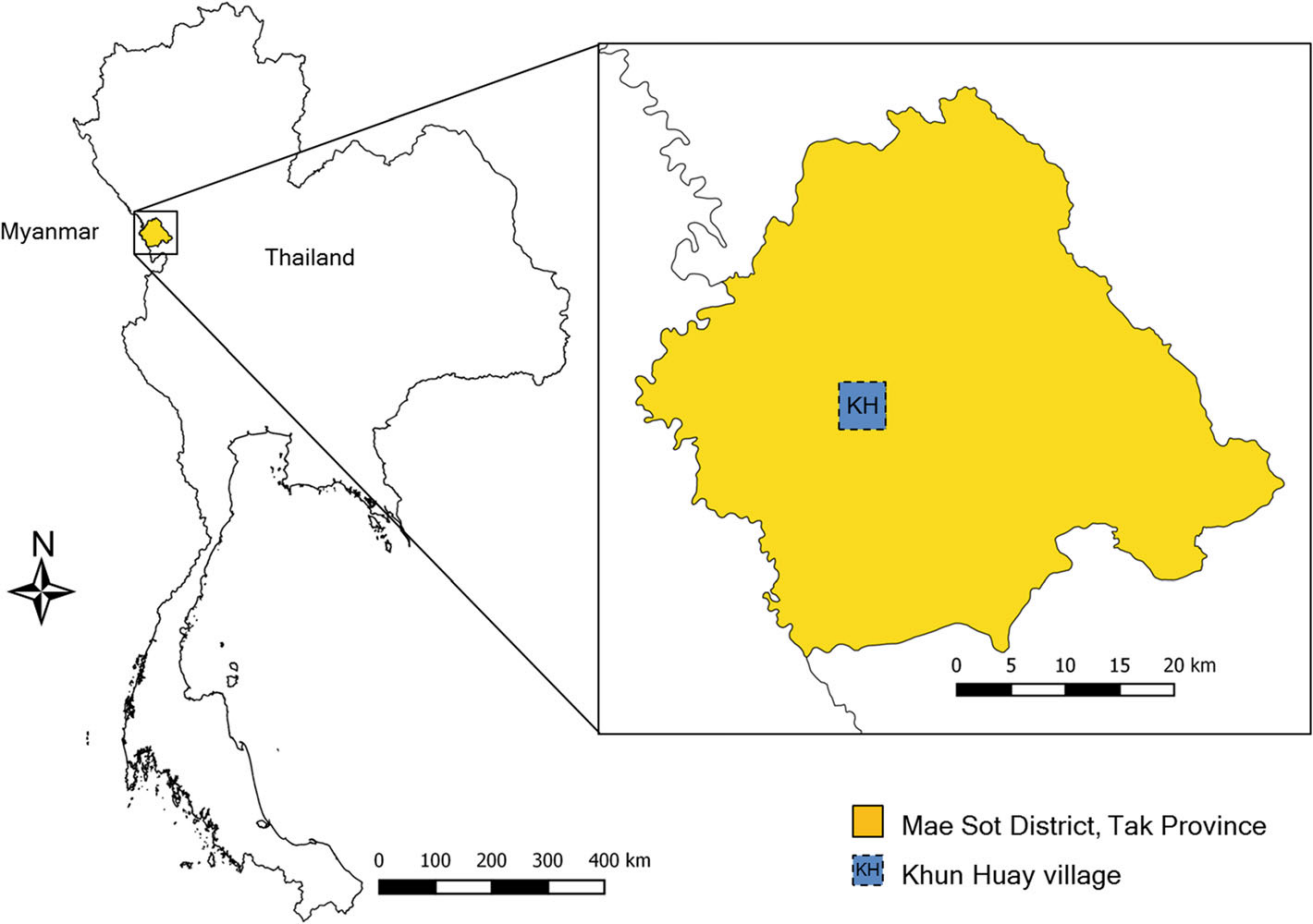
The study site at Khun Huay village along the Thai-Myanmar boarder in Mae Sot District, Tak Province, Thailand

Trap comparison experiments were carried out in a malaria-endemic area located in Khun Huay, a village of over 100 non-enclosed homes, located in Mae Sot District, Tak Province in northwestern Thailand along the Thai-Myanmar border (16°43′14″N, 98°39′45″E, Fig. 1). Residents are either Thai or Karen (from Myanmar) subsistence farmers of rice, maize, and soybean and rear livestock including cattle, pigs, and goats. The region is characterized by three seasons: rainy (mid-May to mid-September), winter (mid-September to mid-February), and summer (mid-February to mid-May). The current study was carried out from June to December 2010 with mean daily temperatures ranging between 22.4 and 29.8 °C.

### Experimental design

#### Comparative field evaluation of BGS-trap and CDC LT

Field evaluations were carried out using the BGS Trap and CDC LT. Six trap configurations were evaluated using a Latin square design as follows: (i) BGS Trap with BG-lure; (ii) BGS Trap with BG-lure plus CO_2_; (iii) BGS Trap with CO_2_; (iv) CDC LT with BG-lure; (v) CDC LT with BG-lure plus CO_2_; and (vi) CDC LT with CO_2_, respectively. Traps and attractants were operated according to manufacturers’ instructions. Note that for the present study, the BGS Trap did not contain the BG-lure unless specified. Dry ice (1 kg per trap) placed in an insulated plastic container with a release pipe was used as the CO_2_ source.

The village was divided into 6 cluster areas. Within each cluster area, 3 traps were operated for one particular trap configuration from 18:00 to 6:00 h with each trap in the cluster area separated by distances of at least 200 m. Therefore, for one collection night, a total of 18 traps was operated across the 6 clusters representing all 6 configurations. To account for position effects, the 3 traps for one particular configuration were rotated counterclockwise to the adjoining cluster prior to sampling the subsequent night.

Collected mosquitoes were placed on dry ice for subsequent identification. Females were morphologically identified to species [26] and parity was determined.

#### Efficacy evaluation of BGS trap and CDC LT augmented with different colored lights

**Fig. 2:**
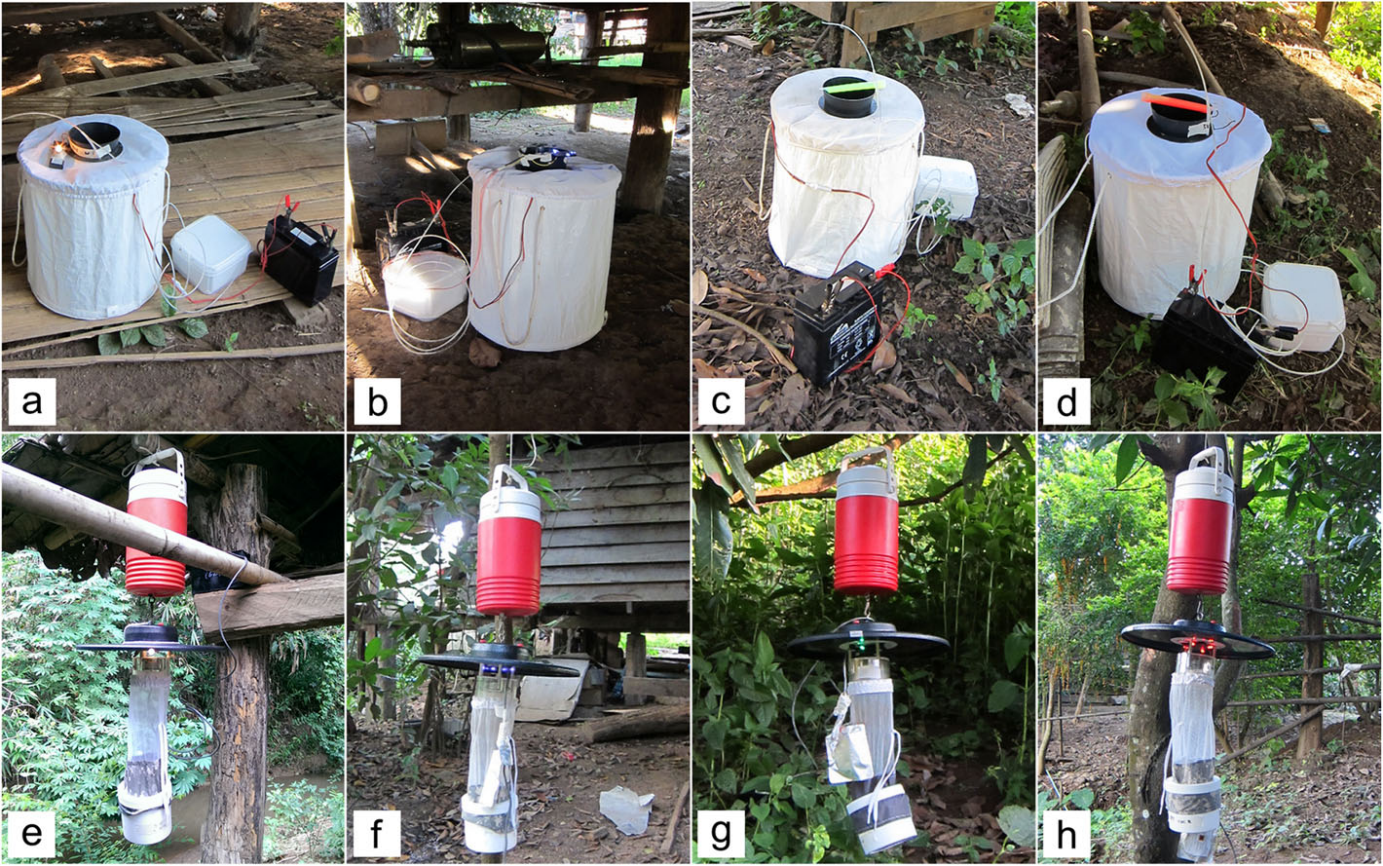
Trap setting. **a** BGS trap with incandescent bulb. **b** BGS trap with UV LED. **c** BGS trap with green light stick. **d** BGS trap with red light stick, I CDC LT with incandescent bulb. **f** CDC LT with UV LED. **g** CDC LT with green LED. **h** CDC LT with red LED

The efficacies of the BGS Trap and CDC LT (both trap types baited with BG-lure plus CO_2_) augmented with different light sources including light bulbs, light sticks and light-emitting diodes (LED) were evaluated for collecting anopheline mosquitoes*.* Four light configurations were evaluated for the BGS Trap: (i) incandescent bulb; (ii) UV LED; (iii) green light stick; and (iv) red light stick. For the CDC LT evaluation, four light configurations were tested: (i) incandescent bulb; (ii) UV LED; (iii) green LED; and (iv) red LED (Fig. 2). The 1.5-watt incandescent light (Chicago Miniature Lighting, Hackensack, NJ) was used as the control in this experiment. Colored LED light bulbs (UV, green, and red) were purchased from the local market in Thailand (Budget LED, Bangkok, Thailand). Four LED bi-pin bulbs of the same color arranged in a circular alignment were connected to each LED trap (Fig. 2). Six-inch green and red chemical light sticks rated for 12 h were used for the BGS Trap’s light source (LC Industries, Durham, NC). The light sticks were waterproof, non-toxic and non-flammable light sources emitting a 360-degree bright light.

The village was divided into two areas; one area was used to evaluate the BG Trap configurations and the other area was used for the CDC LT configurations. Each area was further divided into a set of four cluster areas. Within each cluster area, two traps, separated by approximately 200 m, were operated for one particular light configuration from 18:00 to 6:00 h. Therefore, for one collection night, 16 traps were operated across the 8 clusters representing all 8 configurations. Traps within each of the two areas (BGS Trap or CDC LT) were rotated counterclockwise after each of four collection days, comprising one replicate of the study. Six replicates were conducted. All collected samples were maintained in a cold chain (dry ice) upon transport to the field laboratory. Collected mosquitoes were separated by sex and enumerated. Collected females were morphologically identified to species and the parity rate was determined.

#### Physiological state determination

After identification to species, *Anopheles* females were dissected under a microscope and classified as either nulliparous (coiled tracheole skeins visible in the ovaries), recently blood-fed (blood in the midgut), gravid (ovarioles developed past stage II), or parous (absence of tracheolar skeins in ovaries) [27].

### Data analysis

All statistical analyses were performed using IBM SPSS statistical software, version 23. Total number of *Anopheles* and *An. minimus* Theobald females captured from each trap configuration were pooled by night (either 2 or 3 traps/trap configuration/night) for statistical analysis. Generalized linear model (GLM) with negative binomial error and log link function was used to analyze effects of trap configurations, different attractants and colored lights on the numbers of mosquitoes collected in both experiments. The dispersion coefficients were estimated using maximum likelihood estimation. Parameter coefficients were tested using Wald Chi-square. Incident rate ratios (IRR) of BGS Trap, tested attractants, and colored lights were calculated relative to CDC LT with CO_2_ and incandescent light as the reference. The values of IRR greater or lower than 1 indicate higher or lower trapping performance relative to the reference one. The hypothesis was tested using the Chi-square (*χ* ^2^). The physiological state (parity rate) was determined and calculated as a percentage of parous females from the total mosquitoes dissected. The Chi-square (*χ* ^2^) test was used to statistically analyze the physiological state data.

## Results

### Comparative field evaluation of BGS-trap and CDC LT

A total of 42 mosquito species (6079 female mosquitoes) was collected. From the collections spanning 324 trap-nights, the predominant species were *Cx. vishnui* Theobald (28.66%), *An. minimus* (25.86%) and *Cx. quinquefasciatus* Say (12.22%). The BGS Trap (with CO_2_, BG-lure, or both) and CDC LT (with CO_2_, BG-lure, or both) collected 1676 (27.57%) and 4403 (72.43%) female mosquitoes, respectively (Table 1). The BGS Trap collected a total of 30 mosquito species while the CDC LT collected 38 mosquito species. The predominant species collected by the BGS Trap were *Cx. quinquefasciatus* (*n* = 685), *Cx. vishnui* (*n* = 354), *An. minimus* (*n* = 241), *An. vagus* Doenitz (*n* = 143) and *Ae. albopictus* (*n* = 88). Species most commonly found in the CDC LT included *An. minimus* (*n* = 1340), *Cx. vishnui* (*n* = 1388), *Ae. vexans* (Meigen) (*n* = 395), *An. sawadwongporni* Rattanarithikul & Green (*n* = 162), *An. vagus* (*n* = 162), *Cx. gelidus* Theobald (*n* = 157), *Ae. vigilax* (Skuse) (*n* = 144) and *An. splendidus* Koidzumi (*n* = 119). Notably, the CDC LT trapped more species and greater numbers of anthropophilic malaria vectors such as *An. minimus*, *An. vagus*, *An. sawadwongporni* and *An. splendidus* than the BGS-Trap (Table 1).

**Table 1:** Total numbers of female mosquitoes collected from each tested trap configuration from both experiments conducted in Khun Huay, Mae Sot District, Tak Province, from June to December 2010

	Trap type	Tested attractant	Trap-night	*Anopheles*	*An. minimus*	*Aedes*	*Culex*	Others^a^
Exp. I	CDC LT	CO_2_	54	646	412	347	673	24
		BG-lure + CO_2_	54	973	693	207	999	25
		BG-lure	54	409	230	11	82	7
	BGS Trap	CO_2_	54	204	90	39	457	11
		BG-lure + CO_2_	54	204	132	60	375	16
		BG-lure	54	41	15	16	248	5
Exp. II	CDC LT	Incandescent	48	585	189	2	25	48
		UV LED	48	448	127	10	23	20
		Green LED	48	298	79	6	5	21
		Red LED	48	410	168	6	6	11
	BGS Trap	Incandescent	48	96	34	49	24	31
		UV LED	48	81	12	10	41	73
		Green light stick	48	43	5	27	13	29
		Red light stick	48	27	3	36	26	27

^a^Others include mosquitoes in 5 genera (*Armigeres*, *Downsiomyia*, *Lutzia*, *Uranotaenia* and *Mansonia*)

Statistical analysis revealed that trap type and attractants affected numbers of female anopheline and *An. minimus* collections (Table 2). The performance of the BGS Trap for collecting female anopheline and *An. minimus* was significantly lower than CDC LT (female anopheline: *χ* ^2^ = 60.207, *df* = 1, *P* < 0.0001; *An. minimus*: *χ* ^2^ = 71.333, *df* = 1, *P* < 0.0001) (Table 2). The combination of BG-lure and CO_2_ as the trap attractants tended to enhance anopheline and *An. minimus* collections (IRR = 1.20 and 1.57) albeit not significantly. The addition of BG-lure to the gold standard technique for malaria vector collection (CDC LT with CO_2_) increases the effectiveness of sampling for *Anopheles* (IRR = 1.72, *P* = 0.041) and *An. minimus* (IRR = 1.99, *P* = 0.013) (Table 3). Interestingly, comparison of efficacies (*Anopheles* collection) between CDC LT baited with either CO_2_ or BG-lure alone revealed no statistically significant differences (IRR = 0.67, *P* = 0.135). The current field study shows that the BG-lure, when used in concert with CO_2_, can significantly enhance *Anopheles* and *An. minimus* CDC LT collections relative to the CDC LT with just CO_2_.

**Table 2:** The efficacy of the BGS trap and different attractants relative to the CDC LT and CO_2_ for collecting anophelines and *An. minimus*

	Treatment	IRR	95% CI	*χ* ^2^	*P*-value
♀ Anopheline	CDC light trap (reference)				
	BG-sentinel	0.19	0.13–0.29	60.21	< 0.0001
	CO_2_ (reference)				
	BG-lure + CO_2_	1.20	0.74–1.97	0.54	0.462
	BG-lure	0.38	0.23–0.63	13.80	< 0.0001
♀ *An. minimus*	CDC light trap (reference)				
	BG-sentinel	0.15	0.10–0.23	71.33	< 0.0001
	CO_2_ (reference)				
	BG-lure + CO_2_	1.57	0.94–2.61	2.98	0.084
	BG-lure	0.36	0.21–0.61	13.93	0.002

*Abbreviations: IRR* estimated incident rate ratio, *CI* confidence interval, corresponding *P*-value based on maximum likelihood estimation of GLM with negative binomial error and log link function

**Table 3:** The efficacy of different attractants (for each trap type) relative to the CO_2_ for collecting anophelines and *An. minimus*

		Treatment	IRR	95% CI	*χ* ^2^	*P*-value
CDC LT	♀ Anopheline	CO_2_ (reference)				
		BG-lure + CO_2_	1.72	1.02–2.88	4.19	0.041
		BG-lure	0.67	0.40–1.13	2.23	0.135
	♀ *An. minimus*	CO_2_ (reference)				
		BG-lure + CO_2_	1.99	1.16–3.41	6.19	0.013
		BG-lure	0.63	0.36–1.09	2.74	0.098
BGS-Trap	♀ Anopheline	CO_2_ (reference)				
		BG-lure + CO_2_	1.02	0.66–1.60	0.01	0.917
		BG-lure	0.19	0.11–0.33	34.55	< 0.0001
	♀ *An. minimus*	CO_2_ (reference)				
		BG-lure + CO_2_	1.55	1.01–2.39	4.00	0.046
		BG-lure	0.18	0.09–0.34	27.65	< 0.0001

*Abbreviations: IRR* estimated incident rate ratio, *CI* confidence interval, corresponding *P*-value based on maximum likelihood estimation of GLM with negative binomial error and log link function

Within the BGS Trap collections, we found that augmentation with CO_2_ can improve the efficacy of the trap for malaria vector collections. The number of *Anopheles* collected by BGS Traps augmented with CO_2_ was significantly higher than BGS Trap baited only with BG-lure (*χ* ^2^ = 34.554, *df* = 1, *P* < 0.0001, Table 3). However, the efficacy of BGS-Trap baited with CO_2_ was not significantly different from the trap baited with BG-lure plus CO_2_ for *Anopheles* capture (*P* = 0.92). For *An. minimus* collections, adding BG-lure into the BGS Trap baited with CO_2_ significantly improved the trap efficacy (IRR = 1.55, *P* = 0.046, Table 3).

Age grading and blood meal status identification took place for a total of 2477 *Anopheles* females (Table 4). Almost all of the females were found to be in the empty stage (99.1%). Notably, nearly half of the samples captured by the BGS Trap baited with BG-lure were recently blood-fed specimens (41.5%, *n* = 17). Parity rates of *Anopheles* mosquitoes collected by BGS-Trap and CDC LT baited with different attractants ranged from 31.7 to 44.1%. No significant differences could be detected (*χ* ^2^ = 5.79, *P* = 0.33, Table 4).

**Table 4:** Percentage of female *Anopheles* (actual number) in different physiological conditions collected from each trap configuration and the parity rate for each trap configuration

Physiological condition	BGS-Trap			CDC LT			Grand total
	BG-lure	BG-lure + CO_2_	CO_2_	BG-lure	BG-lure + CO_2_	CO_2_	
Nulliparous	19.5 (8)	55.4 (113)	63.7 (130)	64.6 (264)	62.9 (612)	64.1 (414)	62.2 (1541)
Parous	31.7 (13)	44.1 (90)	35.8 (73)	35.5 (145)	37.0 (360)	35.9 (232)	36.9 (913)
Gravid	7.3 (3)	0.5 (1)	0.5 (1)	0 (0)	0 (0)	0 (0)	20.2 (5)
Recently blood-fed	41.5 (17)	0 (0)	0 (0)	0 (0)	0 (1)	0 (0)	0.7 (18)
Total	41	204	204	409	973	646	2477
Parity rate (%)	31.7	44.1	35.8	35.5	37.0	36.0	

### Efficacy evaluation of BGS trap and CDC LT augmented with different colored lights

For over 384 trap-nights, a total of 2557 female mosquitoes were captured representing 41 species in 11 genera including *Anopheles*, *Armigeres*, *Culex*, *Aedes*, *Bothaella*, *Lorrainea*, *Downsiomyia*, *Malaya*, *Mansonia*, *Uranotaenia* and *Heizmannia* (Table 1). *Anopheles* spp. were the most abundant (77.7% of the total collected mosquitoes, *n* = 1988). Seventeen *Anopheles* species were collected of which *An. minimus* was predominant (*n* = 1490). The BGS-Trap (all configurations) and CDC LT (all configurations) trapped 633 and 1924 mosquito females, respectively (Table 1). The main species collected by the BGS Trap were *An. minimus* (*n* = 164), *Ar. subalbatus* (Coquillett) (*n* = 137), *Ae. albopictus* (*n* = 100), *Cx. vishnui* (*n* = 85) and *An. peditaeniatus* (Leicester) (*n* = 55) while the common species collected by CDC LT were *An. minimus* (*n* = 1326), *An. peditaeniatus* (*n* = 276), *Ar. subalbatus* (*n* = 89) and *An. tessellatus* Theobald (*n* = 44). All totaled, a greater number of anophelines was attracted to the CDC LT configurations than the BGS Trap configurations.

The trap type, and different colored lights significantly influenced the number of female anophelines (GLM, *χ* ^2^ = 130.261, *df* = 1, *P* <0.0001 and *χ* ^2^ = 13.082, *df* = 3, 0.004, respectively) and the number of *An. minimus* collected (GLM, *χ* ^2^ = 120.678, *df* = 1, *P* < 0.0001 and *χ* ^2^ = 12.237, *df* = 3, 0.007, respectively). Significantly lower numbers of female anophelines and *An. minimus* were collected by the BGS Trap relative to the CDC LT (Table 5). None of the colored lights tested could exceed the performance of incandescent bulbs as conventional light sources for CDC LT (Table 5). An overview of the results showed the order of trap efficiency as incandescent > UV > green > red. As expected, the number of female anopheline (IRR = 0.50, *P* = 0.004) and *An. minimus* (IRR = 0.43, *P* = 0.002) collected were significantly lower when traps were augmented with red light (Table 5).

**Table 5:** Efficacy of BGS trap and different colored lights relative to the CDC LT and the incandescent light for capturing anophelines and *An. minimus* females

	Treatment	IRR	95% CI	*χ* ^2^	*P*-value
♀ Anopheline	CDC light trap (reference)				
	BG-sentinel	0.13	0.09–0.19	130.26	< 0.0001
	Incandescent (reference)				
	UV (10–400 nm wavelength)	0.80	0.50–1.29	0.83	0.363
	Green (490–570 nm wavelength)	0.48	0.30–0.77	9.12	0.003
	Red (620–780 nm wavelength)	0.50	0.31–0.81	8.09	0.004
♀ *An. minimus*	CDC light trap (reference)				
	BG-sentinel	0.11	0.08–0.17	120.68	< 0.0001
	Incandescent (reference)				
	UV (10–400 nm wavelength)	0.81	0.48–1.37	0.61	0.436
	Green (490–570 nm wavelength)	0.51	0.30–0.87	6.14	0.013
	Red (620–780 nm wavelength)	0.43	0.25–0.74	9.37	0.002

*Abbreviations: IRR* estimated incident rate ratio, *CI* confidence interval, corresponding *P*-value based on maximum likelihood estimation of GLM with negative binomial error and log link function

Within the CDC LT group, significantly fewer anophelines were collected when green (IRR = 0.56, *P* = 0.001) and red lights (IRR = 0.56, *P* = 0.001) were used as light sources when compared to the numbers collected by the control (incandescent light). Significantly fewer *An. minimus* were collected when green (IRR = 0.55, *P* = 0.001,) and red lights (IRR = 0.57, *P* = 0.002) were used as light sources when compared to the numbers collected by the control (Table 6). However, analysis of the results demonstrated lower catch rates of anopheline females (IRR = 0.74) and *An. minimus* (IRR = 0.81) in the UV light relative to the incandescent light, although no significant differences were statistically detected.

**Table 6:** Efficacy of different colored lights (for each trap type) relative to the incandescent light for collecting anophelines and *An. minimus*

		Treatment	IRR	95% CI	*χ* ^2^	*P-value*
CDC LT	♀ Anopheline	Incandescent (reference)				
		UV (10–400 nm wavelength)	0.74	0.53–1.03	3.11	0.078
		Green (490–570 nm wavelength)	0.56	0.40–0.79	10.99	0.001
		Red (620–780 nm wavelength)	0.56	0.40–0.78	11.54	0.001
	♀ *An. minimus*	Incandescent (reference)				
		UV (10–400 nm wavelength)	0.81	0.56–1.15	1.42	0.233
		Green (490–570 nm wavelength)	0.55	0.38–0.79	10.38	0.001
		Red (620–780 nm wavelength)	0.57	0.40–0.82	9.27	0.002
BGS Trap	♀ Anopheline	Incandescent (reference)				
		UV (10–400 nm wavelength)	0.66	0.38–1.13	2.28	0.131
		Green (490–570 nm wavelength)	0.27	0.14–0.51	16.4	< 0.0001
		Red (620–780 nm wavelength)	0.31	0.16–0.58	13.05	< 0.0001
	♀ *An. minimus*	Incandescent (reference)				
		UV (10–400 nm wavelength)	0.54	0.28–1.04	3.43	0.064
		Green (490–570 nm wavelength)	0.22	0.10–0.48	14.35	< 0.0001
		Red (620–780 nm wavelength)	0.17	0.07–0.40	16.60	< 0.0001

*Abbreviations: IRR* estimated incident rate ratio, *CI* confidence interval, corresponding *P*-value based on maximum likelihood estimation of GLM with negative binomial error and log link function

A similar significant effect of different light colors was observed within the BGS Trap configurations. No significant differences were detected in UV light augmented traps compared to the control (*P* > 0.05, Table 6). However, significantly lower capture numbers of malaria vectors in green and red colored lights relative to the incandescent light were detected (*P* < 0.001).

The physiological stage and blood meal status were determined for 1988 female *Anopheles* collected from the eight trap configurations. Most of the collected females were in the empty stage (98.3%). Only 1.0–3.1% of adults collected from these traps contained blood. Parity rates of *Anopheles* mosquitoes collected by BGS-Trap and CDC LT baited with different colored-lights did not vary significantly, ranging from 37.2–55.6% (*χ* ^2^ = 3.65, *P* = 0.82) (Table 7).

**Table 7:** Percentage of *Anopheles* female (actual number) in different physiological conditions captured from 8 different colored-light trap configurations and the parity rate for each trap configuration

Physiological condition	BGS-Trap				CDC LT				Grand total
	Incandescent	UV LED	Green light stick	Red light stick	Incandescent	UV LED	Green LED	Red LED	
Nulliparous	55.2 (53)	49.4 (40)	62.8 (27)	44.4 (12)	51.8 (303)	51.8 (232)	55.7 (166)	54.9 (225)	53.2 (1058)
Parous	43.8 (42)	48.2 (39)	37.2 (16)	55.6 (15)	46.5 (272)	45.1 (202)	43.3 (129)	44.2 (181)	45.1 (896)
Gravid	0 (0)	0 (0)	0 (0)	0 (0)	0 (0)	0 (0)	0 (0)	0 (0)	0 (0)
Recently blood-fed	1.0 (1)	2.5 (2)	0 (0)	0 (0)	1.7 (10)	3.1 (14)	1.0 (3)	1.0 (4)	1.7 (34)
Total	96	81	43	27	585	448	298	410	1988
Parity rate (%)	43.8	48.2	37.2	55.6	46.5	45.1	43.3	44.2	

## Discussion

The present study represents the first field evaluation of the BGS Trap for capturing malaria vectors in Thailand and southeast Asia. Our findings demonstrated that the BGS Trap captured a variety of mosquitoes (30 species) during the night including important malaria vectors. However, results from this field study are similar to previous studies in which the CDC LT trapped significantly more mosquitoes and anopheline females than the BGS Trap and proved that mosquito species composition depends on trap type [18, 28, 29]. Moreover, important anthropophilic malaria vectors were caught by CDC LT. In addition to the design of the trap, trap height tends to play an important role in mosquito collections [30, 31].

Findings from the present study confirmed that CDC LT, when baited with BG-lure and CO_2_, yields higher numbers of anophelines and *An. minimus* than other trap configurations. Our results parallel a similar environmental chamber study which found that the combination of the BG-lure and CO_2_ proved to be most effective at attracting *An. gambiae* [21]. Surprisingly, our results demonstrated no significant differences in anopheline catch numbers between the CDC LT baited with CO_2_ and the CDC LT baited with the BG-lure alone. Our findings revealed that the BG-lure can be used as a substitute for CO_2_ as the attractant for CDC LT in malaria-centric areas and in study sites where viable CO_2_ sources present a challenge. Our research has shown an equal proportion of parous females in each trap configuration suggesting that physiological stage does not make *Anophele*s prone to one particular trap.

The second objective of this study was to evaluate the efficacy of the BGS Trap and CDC LT when baited with different colored lights for capturing malaria vectors. Findings from the current study show the CDC LT (baited with BG-lure and CO_2_) augmented with an incandescent light is a favorable tool for malaria vector surveillance in Thailand and can be applied in other countries. Interestingly, not all insect visible lights attract *Anopheles* mosquitoes. Our results show that colored lights and trap type have no significant impact on the collections of different physiological stages of female *Anopheles* (both parous and nulliparous). The CDC LT augmented with an incandescent light bulb, BG-lure and CO_2_ is most effective and reliable in collecting anophelines and can be effectively used as the new gold standard technique to collect malaria vectors*.*

## Conclusion

The CDC LT augmented with an incandescent light bulb, BG-lure and CO_2_ is most effective and reliable in collecting anophelines and can be effectively used as the new gold standard technique to collect malaria vectors*.* The BG-lure can be substituted for CO_2_ as an attractant in CDC LT in hard-to-reach malaria sites where CO_2_ sources are scarce. The BGS Trap can collect a variety of night-time feeding mosquitoes including important malaria vectors; however, the number of *Anopheles* collected is significantly lower than the CDC LT. The data presented here will assist researchers in selecting the most appropriate malaria vector surveillance tools.

## Abbreviations

BGS: BG Sentinel™ trap
CDC LT: Centers for disease control and prevention miniature light trap
GLM: Generalized linear model
GMS: Greater Mekong Subregion
HLC: Human landing counts
IRR: Incident rate ratios
LED: Light-emitting diodes
